# Ellipsometric spectroscopy of rubidium vapor cell at near-normal incidence

**DOI:** 10.1038/s41598-020-74255-x

**Published:** 2020-10-13

**Authors:** M. Mosleh, M. Ranjbaran, S. M. Hamidi, M. M. Tehranchi

**Affiliations:** 1 Laser and Plasma Research Institute, Shahid Beheshti University, Tehran, Iran; 2 Department of Physics, Faculty of Science, Central Tehran Branch, Islamic Azad University, Tehran, Iran; 3 Physics Department, Shahid Beheshti University, Tehran, Iran

**Keywords:** Applied optics, Optical techniques, Atomic and molecular interactions with photons, Atom optics

## Abstract

Various efforts have been made to overcome Doppler broadening in hyperfine measurement limitations in the atomic vapors spectroscopy and associated applications. The present study measured and calculated hyperfine resolved ellipsometric parameters through the near-normal reflectance spectra of the rubidium vapor cell in two experimental setups based on continuous and modulated pathway. The results indicated that valuable information could be extracted from the ellipsometric parameters about the atomic medium. Change in the ellipsometric parameters in each transition line confirms the existence of the elliptical polarization of the reflected light when it is exposed to the alkali metal vapor. Our results show that the ellipticity at ^5^S_1/2_ (F_g_ = 1, 2) → ^5^P_1/2_ (F_e_ = 1, 2) hyperfine transitions of ^87^Rb (D_1_ line) is small, and accordingly hyperfine transitions between the ground ^5^S_1/2_ (F_g_ = 2, 3) and excited ^5^P_1/2_ (F_e_ = 2, 3) states of the ^85^Rb isotope are considerable. These ellipsometric parameters, as phase difference, can trace the behavior of the relative orientation of the electric field and atom velocity in the interface based on van der Waals dipole–dipole interaction and is directly proportional to the strength of the light-matter interaction which extremely useful instead complicated atomic spectroscopic methods.

## Introduction

During recent years, fabricating chip-scale atomic devices has attracted a lot of attention. These devices are typically based on micro-fabricated alkali vapor cells^1^. However, the size decrease of the cells is followed by the increment of atom-wall interactions, leading to the deterioration of measurement sensitivity. Accordingly, considering light-matter interactions at atomic dimensions is an alternative solution for overcoming the trade-off between the sensitivity and size of the device. This solution can be an important issue in various applications such as atomic clocks^2^, atomic magnetometers^3–6^, and atomic plasmonic sensors^7^.

Some studies used optical methods such as reflection spectroscopy^8^, evanescent wave spectroscopy^9^, and Doppler-free selective reflection spectroscopy^10^ to evaluate light-matter interactions at the interface of gas-cell walls. By using the above-mentioned methods, main information can be obtained regarding the typical signature of the atomic vapor such as hyperfine transitions on short- and long-range surface interactions. In these common methods, such transitions cannot be distinguished in some frequency regions due to their overlap. So far, it is considered as an open question to find better and accurate methods for delivering the language of atomic vapor cells.

Until now, to overcome this bottleneck for the optical study of processes near the surface and thus distinct all possible hyperfine transitions, buffer gases were added to the atomic vapor cell in order to reduce the collisional and Doppler broadening. This led to signal enhancement by increasing the laser-atom interaction time in addition to narrowing the atomic resonance line-width and thus the increment of the spectral resolution^11,12^. In addition, the use of miniaturized or nanometric atomic ensembles accompanying selective reflection spectroscopy are very useful to enhance the signal-to-noise ratio of light–matter interaction^13,14^.

As another possible way to enhance resolution, there is another basic aspect of ellipsometric spectroscopy, which is an optical technique for evaluating the dielectric properties of the targeted sample by recording its reflection under p- and s-polarized incident lights in different media like as the atomic vapor cell.

In this method, the recorded results of the reflection near-normal incidence were analyzed using highly-resolved ellipsometry parameters of Δ and Ψ. In addition, ellipsometry supplies including valuable non-invasive amplitude and phase information were utilized, which could determine effective dielectric functions and their relationships with nanostructure materials, and in the present case, the dielectric characteristics of the system from reflection spectroscopy^15^. In general, this technique can be used to characterize roughness, composition, thickness (depth)^16^, neural activity^17^, thermoplasmonic^18^, magneto-optical activity^19^, and the like. The measured signal is the change in s- and p-polarizations of the reflectance light which interacts with the material structure of interest. The changes of polarization are quantified by the amplitude ratio (Ψ) and the phase difference (Δ). Ellipsometry has been used as a contact-free instrument for determining the optical properties of a targeted sample since the signal relies on the thickness and material properties^20^. Based on these capabilities of ellipsometric data, we use them to record hyperfine transitions in rubidium (Rb) vapor gas in the glass cell for the first time. Base on the phase-sensitive experimental setups and also calculation, we want to approve the fact that phase can open new insights into the atomic area by different dipole–dipole interaction in the interface of the atom and the cell for altered polarizations in ellipsometric parameters.

## Ellipsometric spectroscopy

**Figure 1:**
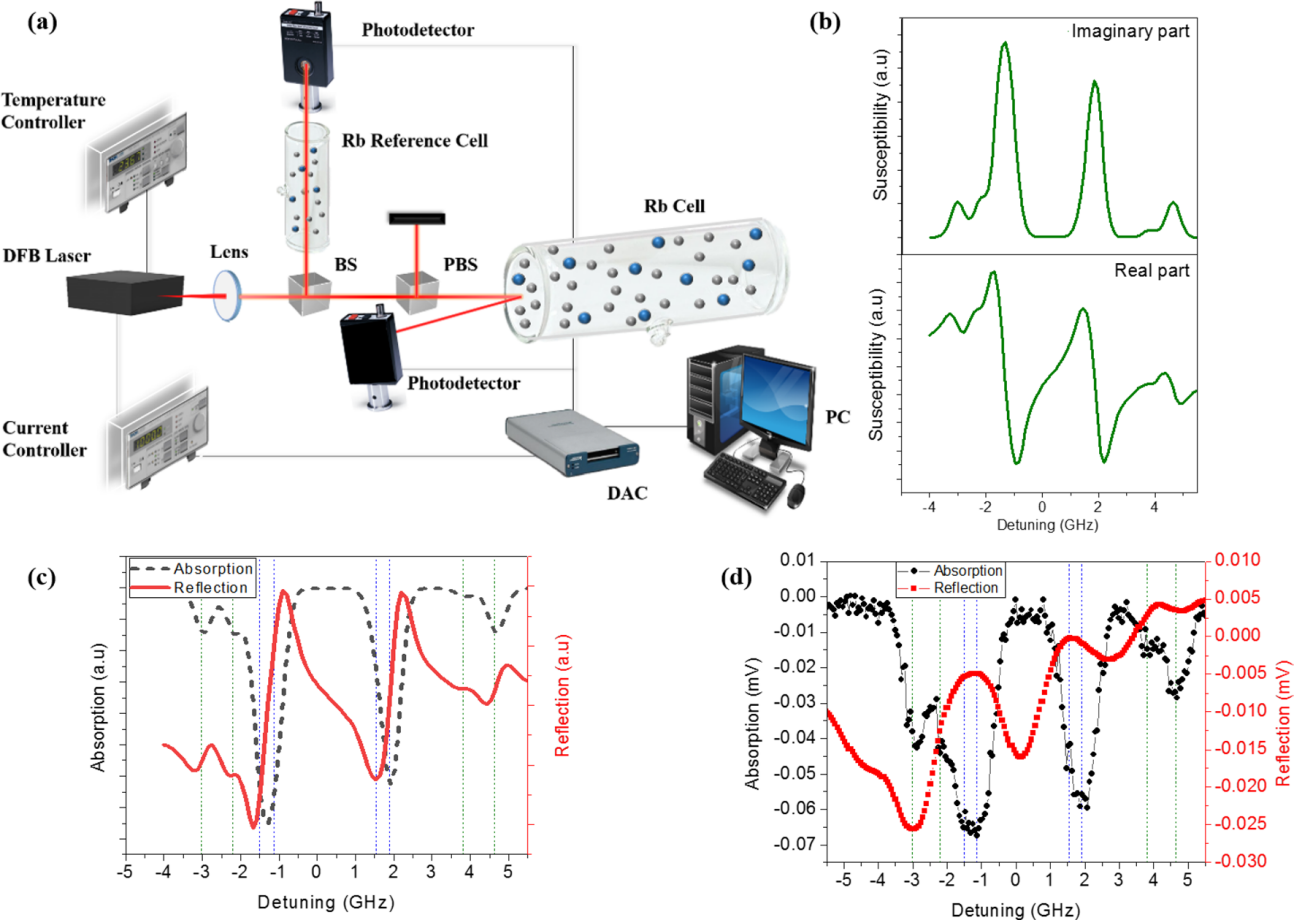
(**a**) Schematic diagram of the experimental setup. (**b**) Calculated real (down) and imaginary (up) parts of Rb susceptibility at 41 °C and 85 MHz Broadening. (**c**) Calculated transmission (black dash-) and reflection (red line) spectrum of Rb vapor cell. The colored Dashed lines show the hyperfine transitions of ^85^Rb and ^87^Rb. (**d**) Experimental absorption (black spheres) and reflection (red squares) spectrum of Rb vapor cell.

The schematic diagram of our experimental setup is shown in Fig. 1a. The light source was a distributed feedback (DFB) laser which was oscillated at 794.8 nm on the D_1_ transition of Rb atoms. To obtain transmission and reflection spectrums, the laser wavelength was scanned across hyperfine transitions by varying its driving current. The laser current was then adjusted by a voltage sweep applied to the laser current controller while its temperature was kept constant. Further, the light beam was split into two separate beams. One beam passed through an Rb reference cell to obtain the transmission spectrum and the other one passed through a Glan-Taylor prism and was divided into s- and p-polarized beams. The s- or p-polarized beam reflected from the surface of our main cell.

The main cylindrical (2 × 5 cm) Pyrex glass cell included natural Rb (comprising 72.2 and 27.8% of ^85^Rb and ^87^Rb isotopes, respectively) with 550 and 10 Torr of He and N_2_ as buffer gases, respectively. Next, the Rb vapor cell was placed in an oven and heated about 41 °C with an electric heater. The transmitted and reflected lights reached large-area photodetectors which were connected to a data acquisition (DAQ) device. Then, the DAQ system measured the changes in output light intensity while the laser wavelength was scanning. All measurement instruments were controlled via USB interfaces by a LabVIEW program.

The reflection spectrum of both s- and p-polarized light beams from the surface of our main Rb cell was required to calculate ellipsometric data. In reflection measurements, the incidence light made an angle of 10° with the surface of the Rb cell. Furthermore, assuring that the reflected light coming from the glass-Rb vapor interface was important since several interfaces and reflections from the boundaries were present. Therefore, all reflected lights were checked and banned except for the reflection from the glass–Rb vapor interface.

Given that the reflected light is elliptically polarized and the parameters of the polarization ellipse are determined by applying the optical constants of both media, using these measured s- and p-polarization components (R_s_ and R_p_), the ellipsometric angles as Ψ and Δ can be extracted as follows.

1 $$\rho = \frac{{r_{p} }}{{r_{s} }} = \tan \psi \exp \left( {i\Delta } \right)$$

where $r_{s,p}$ is Fresnel’s coefficients for *s* and *p* polarizations which should be extracted from the measured reflectance (R_p,s_) in terms of the amplitude and phase as $r\left( \omega \right){\exp}\left( {i\theta \left( \omega \right)} \right)$ in a wide range of frequencies.

2 $$R_{s} = \left| {r_{s} } \right|^{2} = \left| {\frac{{n_{1} \cos \theta_{1} - n_{s}^{2} n_{2} \cos \theta_{2} }}{{n_{1} \cos \theta_{1} + n_{s}^{2} n_{2} \cos \theta_{2} }}} \right|^{2} ,$$

3 $$R_{p} = \left| {r_{p} } \right|^{2} = \left| {\frac{{n_{1} \cos \theta_{1} - n_{p}^{2} n_{2} \cos \theta_{2} }}{{n_{1} \cos \theta_{1} + n_{p}^{2} n_{2} \cos \theta_{2} }}} \right|^{2} ,$$

where n_s_ = 1 and n_p_ = n_1_/n_2_. In this equation, n_1_ and n_2_ are the refractive indices of glass and alkali vapor. Furthermore, θ_1_ and θ_2_ represent the angles of the incidence and refraction, respectively.

Using $R_{s,p} = \left| {r_{s,p} \cdot r_{s,p}^{*} } \right|$, we have

4 $$\left| {r_{s,p} } \right| = \sqrt {R_{s,p} } \to \tan \Psi = \frac{{\sqrt {R_{p} } }}{{\sqrt {R_{s} } }} \to \psi = \tan^{ - 1} \sqrt {\frac{{R_{p} }}{{R_{s} }}} ,\Psi \in \left\langle {0,\left. \pi \right\rangle } \right..$$

So far, Ψ has been calculated easily. To calculate Δ, the following equation was obtained by applying the dependency on the incident light frequency.

5 $$\begin{gathered} R_{s,p} (\omega ) = \left| {r_{s,p} (\omega )r^{*}_{s,p} (\omega )} \right|, \hfill \\ r_{s,p} (\omega ) = \left| {r_{s,p} (\omega )} \right|e^{{i\theta_{s,p} (\omega )}} , \hfill \\ r_{s,p} (\omega ) = \sqrt {R_{s,p} } e^{{i\theta_{s,p} (\omega )}} . \hfill \\ \end{gathered}$$

By using the natural logarithm, we have:

6 $$ln\left[ {r_{s,p} \left( \omega \right)} \right] = ln\left[ {\sqrt {R_{s,p} \left( \omega \right)} } \right] + i\theta_{s,p} \left( \omega \right).$$

Thus, we have a complex function. Hereafter, we apply the concept of Kramers–Kronig relations, which are mathematical relations that are frequently used to connect real and imaginary parts of a complex response function of physical systems by the Hilbert transform. This operation needs some preconditions available in most textbooks^4^. Real and imaginary parts can be related by Eqs. (7) and (8) if we have a physical quantity at specific frequency $\omega_{0}$ as $\alpha \left( {\omega_{0} } \right) = \alpha_{r} \left( {\omega_{0} } \right) + i\alpha_{i} \left( {\omega_{0} } \right)$.

7 $$\alpha_{r} \left( \omega \right) = \frac{2}{\pi }\mathop \smallint \limits_{0}^{\infty } \frac{{\omega^{\prime}\alpha_{i} \left( {\omega^{\prime}} \right) - \omega \alpha_{i} \left( \omega \right)}}{{\omega^{{\prime}{2}} - \omega^{2} }}d\omega^{\prime}$$

8 $$\alpha_{i} \left( \omega \right) = - \frac{2\omega }{\pi }\mathop \smallint \limits_{0}^{\infty } \frac{{ \alpha_{r} \left( {\omega^{\prime}} \right) - \alpha_{r} \left( \omega \right)}}{{\omega^{{\prime}{2}} - \omega^{2} }}d\omega^{\prime}.$$

Therefore, the imaginary parts or the phase of Eq. (5) are calculated as follows.

9 $$\theta \left( \omega \right) = - \frac{2\omega }{\pi }\mathop \smallint \limits_{0}^{\infty } \frac{{ ln\sqrt {R\left( {\omega^{\prime}} \right)} - ln\sqrt {R\left( \omega \right)} }}{{\omega^{{\prime}{2}} - \omega^{2} }} d\omega^{\prime} = - \frac{2\omega }{\pi }\mathop \smallint \limits_{0}^{\infty } \frac{{ ln\sqrt {R\left( {\omega^{\prime}} \right)} }}{{\omega^{{\prime}{2}} - \omega^{2} }} d\omega^{\prime} + \theta_{0} \left( \omega \right)$$

where $\theta_{0}$ is defined as follows.

10 $$\theta_{0} \left( \omega \right) = - \frac{2\omega }{\pi }ln\sqrt {R\left( \omega \right)} \mathop \smallint \limits_{0}^{\infty } \frac{ 1}{{\omega^{{\prime}{2}} - \omega^{2} }} d\omega^{\prime}$$

Additionally, $\Delta$ is defined as the phase difference between *p* and *s* polarizations. Thus, we have:

11 $$\Delta = \theta_{p} \left( \omega \right) - \theta_{s} \left( \omega \right) = - \frac{2\omega }{\pi }\mathop \smallint \limits_{0}^{\infty } \frac{{ ln\sqrt {R_{p} \left( {\omega^{\prime}} \right)} - ln\sqrt {R_{s} \left( {\omega^{\prime}} \right)} }}{{\omega^{\prime 2} - \omega^{2} }} d\omega^{\prime} + [\theta_{0p} \left( \omega \right) - \theta_{0s} \left( \omega \right)] = - \frac{2\omega }{\pi }\mathop \smallint \limits_{0}^{\infty } \frac{{\ln \left( {\sqrt {\frac{{R_{p} \left( {\omega^{\prime}} \right)}}{{R_{s} \left( {\omega^{\prime}} \right)}}} } \right)}}{{\omega^{\prime 2} - \omega^{2} }} d\omega^{\prime} + \Delta \theta_{0}$$

where,

12 $$\Delta \theta_{0} = - \frac{2\omega }{\pi } ln\left( {\sqrt {\frac{{R_{p} \left( {\omega^{\prime}} \right)}}{{R_{s} \left( {\omega^{\prime}} \right)}}} } \right)\mathop \smallint \limits_{0}^{\infty } \frac{ 1}{{\omega^{\prime 2} - \omega^{2} }} d\omega^{\prime}$$

As described above, ellipsometric parameters can now be extracted from the measured reflection spectrum.

However, ellipsometric parameters should similarly be calculated from the calculated reflectance data. Thus, the refractive index of the gas medium is needed at the interface according to Eqs. (3) and (4). Accordingly, we theoretically have the reflectance in each polarization and thus simulated ellipsometric parameters if we obtain the refractive index of the medium. To complete this survey, we derive the electric susceptibility spectrum of Rb alkali atoms including all hyperfine transitions.

Now, it is supposed that a medium exists between a solid with the refractive index of $n_{1}$, occupying the half space z < 0 and a gas occupying the half space z > 0. For simplicity, it is further assumed that the absorption of light in the solid can be neglected and the gas is so rarefied that its refractive index differs only slightly from unity. Due to the reflection of the light from the solid–gas interface, the electromagnetic radiation partly penetrates into the gas medium. The penetrated portion of the light into the gas medium induces a polarization on atoms, and this polarization can be a source of the secondary radiation by the atoms. Therefore, the electric field of the reflected light can be written as $E_{r} = E_{r0} + E_{rp}$, where $E_{r0}$ can be calculated from Fresnel equations and is related to the single solid surface. However, E_rp_ is related to the atoms in the vicinity of the solid surface.

The prediction of the behavior of vapor atoms near the glass surface is possible by using Landau and Lifshitz’ equation of the motion of damped driven oscillator and introducing boundary conditions^21^. A simple but fairly realistic assumption is that vapor atoms near the surface continuously interact with the glass wall surface. The process of excitation and de-excitation of atom polarization occurs during these interactions. According to different theoretical and experimental studies on the reflection of the light from the glass–vapor interface, a fascinating phenomenon known as “selective reflection” occurs due to the collisions of atoms with the surface^15^. Selective reflection spectroscopy signal strongly relies on parameters such as the temperature and density of gas atoms. In present study, the electric susceptibility and refractive index were calculated according to the framework of the classical model of the damped oscillator for the induced polarization of atoms by the incoming electromagnetic field. Taking into account the boundary conditions for atoms moving toward the surface and ones departing from the surface makes it possible to model selective reflection to a good extent. The atoms would be adsorbed when they arrive at the surface. Due to atom-surface interactions, their polarization would be quenched. Consequently, desorbed atoms lose their polarization and do not participate in response to atoms at the reflection. So, the contribution of atoms moving away from the surface could be ignored in reflection spectra^21^.

The approximate electric susceptibility of the Rb atoms, including all its hyperfine transitions is given by Eq. (13) as follows,

13 $$\it {\upchi }\left( {\upomega } \right){ = }\frac{{Ne^{2} }}{{4\pi \varepsilon_{0} m_{e} }}\mathop \sum \limits_{{{\text{F,}}\mathop {F^{\prime}, C_{i} }\limits }} \frac{1}{{\omega_{{FF^{\prime}}} }}\mathop \smallint \limits_{ - \infty }^{\infty } d\upsilon_{x} \mathop \smallint \limits_{0 or - \infty }^{\infty } d\upsilon_{z} \frac{{C_{i} f_{FF\prime } W\left( {\upsilon_{x} ,\upsilon_{z} } \right)}}{{2\pi \left( {\omega_{FF\prime } - {\upomega }} \right) - k_{x} \cdot \upsilon_{x} - k_{z} \cdot \upsilon_{z} - i\gamma }}.$$

The lower limit of $\upsilon_{z}$ equals to $- \infty$ in calculating susceptibility of atoms of bulk medium and 0 for atoms near the surface. N, e, and $\varepsilon_{0}$ demonstrate the number density of vapor atoms, electric charge of the electron, and permittivity of the vacuum, respectively. Further, $m_{e}$, $\omega_{{FF^{\prime}}}$, and $f_{F\prime F}$ indicate the mass of the electron, angular frequency, and the oscillator strength of a transition from a level to another in which *F* and $F^{\prime}$ demonstrate the angular momentum of beginning and final levels, respectively. Furthermore, $\upsilon_{x}$ and $v_{z}$ are velocities and $W\left( {\upsilon_{x} ,\upsilon_{z} } \right)$ is the normalized Maxwellian velocity distribution in parallel and normal to the surface, respectively. Additionally, $k_{x}$ and $k_{z}$ represent the momentum of the transverse light in the vapor medium. Eventually, $\gamma_{a}$ is the FWHM of the lineshape including the natural and collisional broadening and $C_{i}$ denotes the ratio of isotopes. It should be noted that Eq. (12) was only written for x- and z-directions due to the symmetry parallel to the surface while neglecting the y direction.

Now, the refractive index can be related to the electric susceptibility described above as $n\left( \nu \right) = \sqrt {1 + \chi (\nu } )$ although it should be rewritten as $n\left( \nu \right) = \sqrt {1 + 3\chi (\nu )/\left( {3 - \chi (\nu } \right))}$ by considering Fresnel local field correction.

The calculated electric susceptibility and refractive index are complex parameters which have real and imaginary parts, each of which represents distinct physical facts to demonstrate the amount of dispersion and absorption, respectively.

Thus, common Fresnel equations which appear as Eqs. (3) and (4) can be used by this refractive index of the vapor medium in order to derive r_s,p_, and finally theoretically ellipsometric parameters by Eqs. (4)–(10).

As mentioned above,$\psi = \tan^{ - 1} \sqrt {\frac{{R_{p} }}{{R_{s} }}}$ and Δ was the phase difference between *R*_*p*_ and *R*_*s*_. To clarify the role of amplitude and phase of *R*_*p*_ and *R*_*s*_ in resolving power of ellipsometric parameters, our experimental setup was changed to Fig. 3a. As shown in this schematic diagram, an optical modulator and also a lock-in amplifier were added to the previous experimental setup (Fig. 1a) for modulation of laser light and detection of output modulated light signal, respectively. The lock-in amplifier measured the amplitude and phase of the S- and P-polarized reflected signals while the laser frequency was scanned across the hyperfine transitions. The difference between the recorded phase of S- and P-polarized reflected signals was investigated as Δ.

## Results and discussion

The spectral dependence of the electric susceptibility ($\chi$) of rubidium (Rb) atoms under the condition specified in the experimental section was calculated based on Eq. (2) by utilizing python coding. The temperature of the Rb gas vapor and the total broadening (γ) were set at 41 °C and 85 MHz, respectively. The calculation included all hyperfine transitions of the D_1_ line of the two isotopes of natural Rb. In all calculated and experimental results, the relative spacing of the hyperfine-resolved spectrum for both Rb isotopes was reported as detuning from a central frequency. Zero detuning for D_1_ transition was set at the frequency of 5s^2^S_1/2_ → 5p^2^P_1/2_ transition in the absence of hyperfine splitting and was equal to 377,107,407.299 MHz. Oscillator strength (*f*_i_) represents the strength of the interaction of the atom and electromagnetic field in a frequency near the resonance frequency of the atom at each transition and can be calculated from the matrix elements of transitions between defined levels. In the present study, the oscillator strengths of the hyperfine transitions of Rb isotopes were set similar to those of^22^. Table 1 presents the frequency detuning and oscillator strengths of transitions from ground states to desired excite states used in our calculations.

**Table 1:** Detuning from central frequency and oscillator strengths of all hyperfine splitting of the D_1_ line of Rb^22^.

^87^Rb				^85^Rb			
Line (F_g_ → F_e_)	Detuning (MHz)	Oscillator strength	$A_{{F,F^{\prime}}}^{rot}$	Line (F_g_ → F_e_)	Detuning (MHz)	Oscillator strength	$A_{{F,F^{\prime}}}^{rot}$
2 → 1	− 3014.644	$${\raise0.7ex\hbox{$5$} \!\mathord{\left/ {\vphantom {5 {18}}}\right.\kern-\nulldelimiterspace} \!\lower0.7ex\hbox{${18}$}}$$	$$\frac{{3\left( {5 + 3p^{2} } \right)}}{{16\left( {1 + p^{2} } \right)}}$$	3** → **2	− 1497.657	$${\raise0.7ex\hbox{${35}$} \!\mathord{\left/ {\vphantom {{35} {81}}}\right.\kern-\nulldelimiterspace} \!\lower0.7ex\hbox{${81}$}}$$	$$\frac{{2\left( {7 + 14p^{2} + 3p^{4} } \right)}}{{18\left( {3 + 10p^{2} + 3p^{4} } \right)}}$$
2 → 2	− 2202.381	$${\raise0.7ex\hbox{$5$} \!\mathord{\left/ {\vphantom {5 {18}}}\right.\kern-\nulldelimiterspace} \!\lower0.7ex\hbox{${18}$}}$$	$$\frac{{5 + 3p^{2} }}{{16\left( {1 + p^{2} } \right)}}$$	3** → **3	− 1135.721	$${\raise0.7ex\hbox{${28}$} \!\mathord{\left/ {\vphantom {{28} {81}}}\right.\kern-\nulldelimiterspace} \!\lower0.7ex\hbox{${81}$}}$$	$$\frac{{10\left( {7 + 14p^{2} + 3p^{4} } \right)}}{{18\left( {3 + 10p^{2} + 3p^{4} } \right)}}$$
1 → 1	3820.046	$${\raise0.7ex\hbox{$1$} \!\mathord{\left/ {\vphantom {1 {18}}}\right.\kern-\nulldelimiterspace} \!\lower0.7ex\hbox{${18}$}}$$	$$\frac{{1 - P^{2} }}{{16\left( {1 + p^{2} } \right)}}$$	2** → **2	1538.063	$${\raise0.7ex\hbox{${10}$} \!\mathord{\left/ {\vphantom {{10} {81}}}\right.\kern-\nulldelimiterspace} \!\lower0.7ex\hbox{${81}$}}$$	$$\frac{{5 - 2p^{2} - 3p^{4} }}{{18\left( {3 + 10p^{2} + 3p^{4} } \right)}}$$
1 → 2	4632.339	$${\raise0.7ex\hbox{$5$} \!\mathord{\left/ {\vphantom {5 {18}}}\right.\kern-\nulldelimiterspace} \!\lower0.7ex\hbox{${18}$}}$$	$$\frac{{ - 5\left( {1 - P^{2} } \right)}}{{16\left( {1 + p^{2} } \right)}}$$	2** → **3	1900.087	$${\raise0.7ex\hbox{${35}$} \!\mathord{\left/ {\vphantom {{35} {81}}}\right.\kern-\nulldelimiterspace} \!\lower0.7ex\hbox{${81}$}}$$	$$\frac{{ - 7\left( {5 - 2p^{2} - 3p^{4} } \right)}}{{18\left( {3 + 10p^{2} + 3p^{4} } \right)}}$$

Figure 1b displays the real and imaginary parts of the calculated susceptibility of Rb vapor as a function of frequency detuning. As shown, the real part of susceptibility has anomalous dispersion-like behavior and the imaginary part has a Lorentzian spectral lineshape with smaller amplitudes compared to the real part. In Fig. 1c, the black dashed line depicts the transmission spectrum through Rb vapor which is calculated based on the Beer-Lambert law by applying the imaginary part of the calculated refractive index as the absorption coefficient. Further, the reflectance spectrum of the *p*-polarized laser light from the glass–vapor interface was calculated using Fresnel equations (Fig. 1c, red curve). In these calculations, we applied $- \infty$ as a the lower limit of υ_z_ in the susceptibility computation. Based on the data on Table 1, hyperfine transitions between the ground state ^5^S_1/2_ (F_g_ = 2, 3) and excited state ^5^P_1/2_ (F_e_ = 2, 3) of ^85^Rb isotope are determined with blue dashed lines. The peaks corresponding to ^5^S^1/2^ (F_g_ = 1, 2) → ^5^P_1/2_ (F_e_ = 1, 2) hyperfine transitions of ^87^Rb (D_1_ line) are presented with green dashed lines as well.

As shown in Fig. 1d, the transmission and reflection spectrums of our Rb vapor cell were measured in accordance with the theoretical part. During the measurements, the temperature of the Rb cell was kept about 41 °C and in agreement with calculations. Figure 1d illustrates the measured transmission (black circles) and reflection (red squares) spectrums, confirming the finely resolved hyperfine splitting. This resolved spectrum is a consequence of adding He as a buffer gas to the Rb vapor which narrows spectral lines. The narrowing effects were believed to be due to Doppler and collisional broadening suppression because of the uniform velocity distribution of atoms in the presence of the buffer gas. Furthermore, the mean free path between their collisions reduced as the Rb atoms diffused slowly through the buffer gas. This reduction led to the increment of the interaction time of Rb atoms with the laser radiation which enhanced the absorption signal and its narrowing effects^11,12^.

The comparison of the measured data in Fig. 1d with the calculated results in Fig. 1c demonstrates a good agreement between the curves. In both figures, the behavior of the reflection spectrum differs from the transmission spectrum and this difference becomes apparent when considering the dispersion-like behavior of reflection at each frequency detuning. This behavior for the transition-resolved reflection spectrum from the atomic glass–Rb vapor interface is completely in line with the theoretical results based on Fresnel relations^23^. The reflected light from the glass surface of Rb cells contains different information from the transmitted light through the cell. The difference stems from the fact that the transmitted and reflected lights interact with atoms, in the cell volume and vicinity of the glass–Rb vapor interface, respectively.

**Figure 2:**
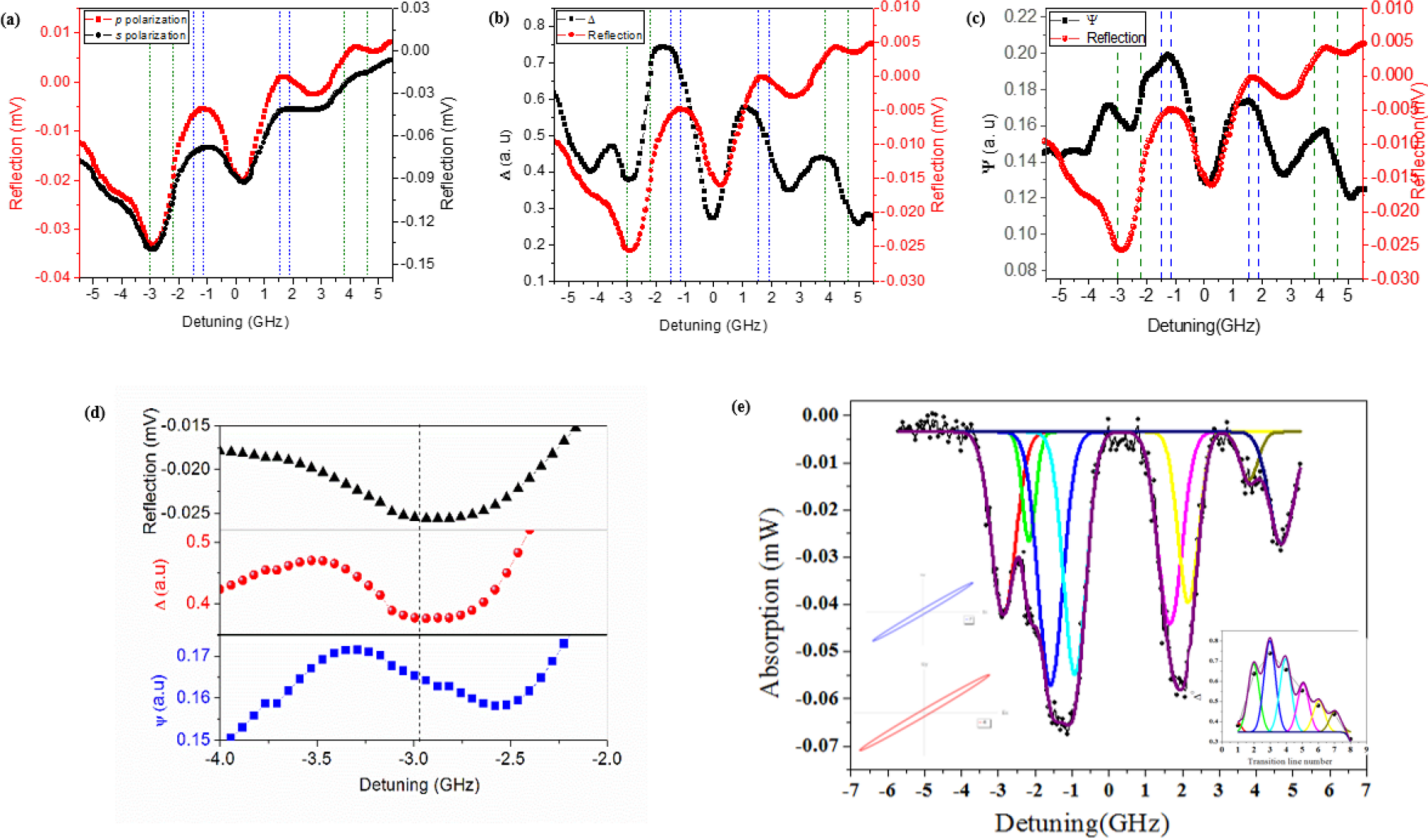
(**a**) Reflection spectrums for S (black spheres) and P (red squares) polarization, Ellipsometry parameters as (**b**) ψ and (**c**) Δ (compared with reflection of p polarization), (**d**) comparison of ψ, Δ and reflection of p polarization at F_g_ = 2 → F_e_ = 1 hyperfine transition (**e**) fitted Lorentzian line-shapes at the position of each transition line of the transmission spectrum (main curve), ellipse of polarization of the reflected light for ^87^Rb (blue line) and ^85^Rb (red line) isotopes (left inset) and Δ variation for each hyperfine transition (right inset).

In contrast to transmission-based measurements, when the laser beam reflects near normally from the glass–vapor interface, it contains local information about the atomic spin polarization very close to the surface over a distance of the order of the optical wavelength^24,25^. Accordingly, the reflection spectrum of the laser beam was measured at both *s* and *p* polarizations from the surface of the Rb cell for collecting ellipsometric parameters. Figure 2a depicts the measured reflection spectrum for *s* (black circles) and *p* (red squares) polarizations. Additionally, the behavior of vapor atoms was considered with respect to different polarization geometries in order to evaluate the origin of a small difference between reflection spectrums for *s* and *p* polarizations. As shown, no difference is observed in the response of Rb atoms to the laser light and the transmission spectra of these polarizations due to the symmetry of the orientation of *s*- and *p*-polarizations in the space. On the other hand, two different groups of atoms are found near the glass surface. One group includes atoms which fly parallel to the glass surface with no wall collisions and thus no interruption in the optical-pumping-induced polarization. The other group encompasses optically polarized atoms moving normally to the glass–vapor interface with frequent wall collisions. These atoms have transient behavior when moving away from the glass–vapor interface until establishing their polarization. Therefore, the wave vector of the incident *s*-polarized light interacts more with the first group of atoms since it is parallel to the interface. However, the parallel and normal components of the *s*-polarized light wave vector interact with both groups of atoms. Thus, the dispersion property of *s*- and *p*-polarized reflected lights becomes different.

One of the other most important interactions is the van der Waals (vW) dipole–dipole interaction between the atomic dipole and its electrostatic image induced in the reflecting surface. The interaction potential which is responsible for the vW attractive force could be expressed as

14 $$V = - \frac{1}{{4\pi \varepsilon_{0} }}\frac{{\left( {d^{2} + d_{z}^{2} } \right)}}{{16z^{3} }},$$

where d is the dipole vector and z indicates the distance between dipole and surface^26,27^. This potential is anisotropic and So the surface effects are different when the electric field applied parallel or perpendicular to the surface.

In the case of P polarization, in which E-field is parallel to the plane of incidence, we have polarization charges and thus the dipole moments parallel to the surface and thus dominant vW interactions, while S polarized incident radiation creates different polarization charges and so different vW dipole–dipole interaction. The difference manifests itself in a frequency shift of P relative to S reflection spectrums.

As shown in Fig. 2b,c, obtaining ellipsometry parameters, the amplitude ratio (ψ), and phase difference (Δ) from the measured reflection spectrums of *s*- and *p*-polarized lights is surprising for further distinguishing between hyperfine transitions. The reflection spectrum of the *p*-polarized light is depicted as a reference in both figures. Based on these parameters, no shift is observed in the spectral position of transition frequencies in both Δ and ψ. In the Δ spectrum, a correspondence is found between the dip and peak in the position of each transition. However, the behavior of the ψ spectrum corresponds to the arctan function of frequency (as shown obviously in Fig. 2d) which is completely in agreement with Eq. (3) in the theoretical section. As expected from the phase difference parameter of Δ, a more distinguishable manner is found for individual transitions mainly in the vicinity of transitions.

The susceptibility and so the refractive index of nonlinear Rb vapor medium heavily relies on frequency detuning^28^. The dependence is investigated in various nonlinear optical effects such as four-wave mixing^12^, self-focusing and self-defocusing^29^. The findings of the present study revealed that the refractive index of Rb vapor and so the reflection from the glass-vapor interface were associated with the frequency detuning for each hyperfine transition due to the frequency detuning dependence of susceptibility, which were demonstrated as the reflection spectrums of *s*- and *p*-polarized lights. Accordingly, ellipsometry data (Δ and ψ) values completely differ for each hyperfine transition.

In other words, when the hyperfine splitting is resolved, the absorption amplitude of each F → F′ transition could be considered individually based on its oscillator strength (as mentioned in Table 1). However, in the ellipsometry technique, the incident light is linearly polarized and we should consider the optical rotation resulting from each of the transitions. The optical rotation, $A_{{F,F^{\prime}}}^{rot}$, as a function of polarization listed in Table 1^30,31^.

The variation of Δ as a function of hyperfine transition numbers is presented in the right inset of Fig. 2e. The transition numbers are defined as 1, 2, 7, 8 for 2 → 1, 2 → 2, 1 → 1, 1 → 2 transitions of ^87^Rb and 3 to 6 for 3 → 2, 3 → 3, 2 → 2 & 2 → 3 transitions of ^85^Rb, respectively. As shown in this figure, the value of Δ increases and decreases based on the relative absorption and optical rotation amplitudes associated with each of transitions F → F′ and also the abundance percentage of each isotope. To clarify the relative absorption of hyperfine structure components, Lorentzian line-shapes were fitted on the position of each transition line of the transmission spectrum, too.

To confirm the results, the polarization ellipse of the reflected light was calculated and drew for two ^87^Rb and ^85^Rb isotopes in the left inset of Fig. 2e. The red bigger and black smaller ellipses belong to ^85^Rb and ^87^Rb isotopes, respectively. The change in ellipsometric parameters in each transition line confirms the existence of the elliptical polarization of the reflected light when it is exposed to the alkali metal vapor. Referring to the above-mentioned theoretical and experimental studies, the ellipticity at ^5^S_1/2_ (F_g_ = 1, 2) → ^5^P_1/2_ (F_e_ = 1, 2) hyperfine transitions of ^87^Rb (D_1_ line) is small, and accordingly hyperfine transitions between the ground ^5^S_1/2_ (F_g_ = 2, 3) and excited ^5^P_1/2_ (F_e_ = 2, 3) states of the ^85^Rb isotope are considerable in other transition lines.

**Figure 3:**
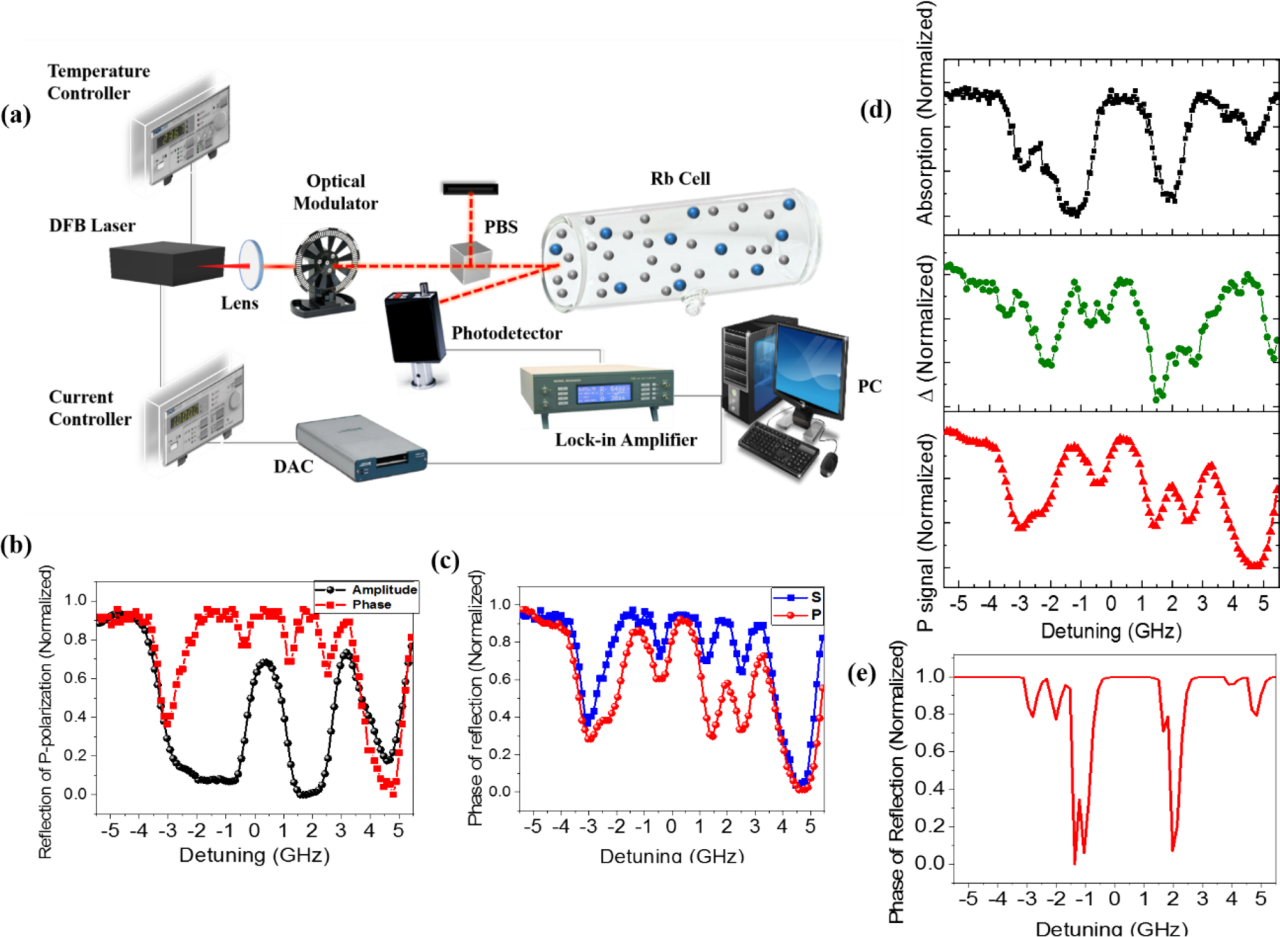
(**a**) Schematic diagram of experimental setup for phase-sensitive detection method. (**b**) The measured amplitude (black circles) and phase (red squares) of reflected P-polarized light. (**c**) The measured phase of reflected S- (blue squares) and P- (red circles) polarized light, (**d**) comparison between the absorption signal (black squares), phase difference between reflected S- and P-polarized light (green circles) and phase of reflected P-polarized light (red triangles). (**e**) The calculated phase of selective reflection spectrum.

At the next step, we utilized a phase-sensitive detection method to elucidate the role of Δ in resolving the hyperfine transitions of atomic rubidium vapor. The recorded amplitude and phase of reflected P-polarized light as a function of laser frequency detuning are depicted in Fig. 3b. One can see that hyperfine transitions are resolved in phase spectrum compared to the amplitude spectrum. To obtain the ellipsometric Δ parameter, we needed the phase difference between R_s_ and R_p_ spectrums. Consequently, the phase spectrums of S- and P-polarized light were recorded and plotted in Fig. 3c.

As mentioned before, atom-wall surface interactions play a critical role in the formation of the reflection signal. The difference between the S and P polarization states is observed in Fig. 3c, clearly. The frequency shift comes from the fact that S- and P-polarized lights cause different vW dipole–dipole interactions at the glass–vapor interface. The difference, defined as Δ parameter, is represented in Fig. 3d (green circles) and compared with absorption and reflection phase spectrums. The resolving power of the ellipsometric Δ parameter is illustrated well in this high-resolution spectrum, while hyperfine transitions are individually distinguished. The higher spectral resolution of this figure relative to Fig. 2b is due to utilizing lock-in amplifier as a phase-sensitive detector.

Furthermore, the phase change of reflected polarized light from the glass-vapor interface was calculated using Fresnel equations (based on Eqs. (2) and (3)). In Fresnel equations, we could write the reflection coefficient as $r = |r|{ }e^{i\theta }$ where $\theta$ is the phase of reflectance. In accordance with the concept of selective reflection, for calculation of phase spectrum, we included transient behavior of atoms departing the surface and exerted 0 as a lower limit of υ_z_ in the computation of the susceptibility. The calculated phase spectrum is depicted in Fig. 3e. It can be seen that individual transitions are well-resolved due to the sub-Doppler structure of selective reflection spectroscopy. Moreover, there is a good agreement between the measured and calculated phase spectrums.

## Conclusion

The present study used ellipsometry as a spectroscopic technique to evaluate reflectance spectra from the surface of the rubidium (Rb) vapor cell. First-order calculations demonstrated a good match with measured data and helped make a proper understanding of the behavior of the systems. The changes in ellipsometric parameters, Ψ and Δ, can be used as valuable tools for studying atom surface interactions since they perfectly trace the physical condition of atoms near the surface. The Δ parameter, the phase difference between S- and P-polarized reflected light, has the power of resolving individual hyperfine transitions well. Our experimental results recorded based on phase-sensitive detection method revealed the resolving power, clearly. The amplitude of Δ at each of hyperfine transitions was related to its relative absorption besides its optical rotation which are functions of oscillator strength and atomic polarization, respectively. Also, our results showed that the frequency shift between the S and P polarization reflection was related to experience different vW dipole–dipole interactions.

In parallel with these measurements, we performed a theoretical analysis by considering the velocity distribution of polarized Rb atoms in parallel and normal to the surface as selective reflection method to illustrate the resolved hyperfine structure in the phase spectrum.

Finally, change in the ellipsometric parameters in each transition line confirms that the ellipticity at ^5^S_1/2_ (F_g_ = 1, 2) → ^5^P_1/2_ (F_e_ = 1, 2) hyperfine transitions of ^87^Rb (D_1_ line) is small, and accordingly hyperfine transitions between the ground ^5^S_1/2_ (F_g_ = 2, 3) and excited ^5^P_1/2_ (F_e_ = 2, 3) states of the ^85^Rb isotope are considerable. Our findings represented that ellipsometry technique provides more spectral resolutions in studying the hyperfine transitions compared to the other complicated spectroscopic methods and can be used for studying the behavior of polarized atoms in vicinity of the glass-alkali vapor interface.
